# An almost fully resolved phylogeny for the Dutch Angiosperm Flora

**DOI:** 10.3897/BDJ.14.e192282

**Published:** 2026-07-23

**Authors:** Thomas Leclère, Andreas Prinzing, Pille Gerhold, Igor Bartish

**Affiliations:** 1 Estonian University of Life Sciences, Tartu, Estonia Estonian University of Life Sciences Tartu Estonia; 2 University of Rennes, Rennes, France University of Rennes Rennes France; 3 Czech Academy of Sciences, Prague, Czech Republic Czech Academy of Sciences Prague Czech Republic

**Keywords:** Molecular phylogeny, Angiosperms, ITS, Plastid markers, GenBank

## Abstract

**Background:**

For several regions, ecologists and taxonomists have assembled information on phenotypes for almost all species of the megadiverse angiosperms. However, testing hypotheses on regional evolutionary and biogeographic history requires highly resolved and dated phylogenies covering the same taxa, which are often lacking at regional scales. Here, we filled this knowledge gap for one of the best-studied regional floras: the native angiosperms of the Netherlands.

**New information:**

We provide a molecular phylogenetic dataset and two time-calibrated trees (based on BEAST and MrBayes approaches) of the native angiosperm flora of the Netherlands, reconstructed from publicly available DNA sequences. The resulting phylogenies include 1178 species from the 2017 species list on SynBioSys NL, a national database that provides a curated checklist of native vascular plant species occurring in the Netherlands (also core to the flora of adjacent countries), and are provided in multiple formats (Newick, Nexus), along with alignments, BEAST and MrBayes input files, and metadata linking taxa to GenBank accession numbers. This dataset offers a phylogenetic framework that is based on molecular data covering more than 95% of the native species included in the checklist, reproducible and extremely well-resolved phylogeny (99.92% of nodes resolved in the maximum clade credibility tree; reflecting the absence of polytomies; 67.9% of nodes with posterior probability above 0.95 and 92.3% above 0.50) for researchers. These data will permit the signal of evolutionary history in patterns of biodiversity across the Netherlands, such as the age structure of habitat species pools or functional groups, where a focus on native species is essential. All resources are openly available via IDiv (https://doi.org/10.25829/idiv.3600-myr3t3) under a CC-BY license.

## Introduction

Understanding biodiversity through the lens of evolutionary history has become a central theme in modern ecology and conservation (Webb et al. 2002, Cavender‐Bares et al. 2009). Phylogenetic trees provide more than just a record of shared ancestry: they are essential tools for quantifying phylogenetic patterns across a native flora, such as identifying evolved species pools of regions or habitat types (Gerhold et al. 2015, Qian et al. 2019, Peterson et al. 2021), tracing biogeographic patterns (Ronquist and Sanmartín 2011), resolving taxonomic issues (The Angiosperm Phylogeny Group 2016), and identifying lineages of high conservation value (Bartish et al. 2020, Hagen 2023). In this sense, robust phylogenetic frameworks are indispensable for linking ecological processes to deep-time evolutionary dynamics (Lamsdell et al. 2017). Although global phylogenies exist for angiosperms (e.g., Zanne et al. 2013, Qian and Jin 2015, Smith and Brown 2018; Baker et al. 2021 or Jin et al. 2026), they often rely on representative taxa, and species from regional floras are often lacking or misidentified. Moreover, such approaches may limit resolution and temporal accuracy for species-level analyses within specific regions (Qian 2019). Highly resolved, dated phylogenies based directly on the species occurring in a focal region are essential for differentiating phylogenetic diversities between patches of the same vegetation type, permitting the inference of conservation priorities of native species (e.g., Hermant et al. 2012, Durka and Michalski 2012, Bartish et al. 2016, Cheikh Albassatneh et al. 2019).

In Europe, and particularly in the Netherlands, angiosperms form the dominant component of terrestrial ecosystems (Ding et al. 2025). The Dutch flora has long been studied from taxonomic and ecological perspectives (Tamis et al. 2005), and extensive species checklists and distributional databases are available. Yet, despite this wealth of information, a comprehensive, up-to-date phylogenetic backbone for the native angiosperms has been lacking. Earlier efforts, such as the Dutch flora phylogeny of Hermant et al. (2012) or the Central European phylogeny (Durka and Michalski 2012), provided important starting points, but they were constrained by limited sequence availability (less than 70% of all native species in the flora), outdated nomenclature, and less flexible analytical approaches. Since then, molecular resources and computational methods have advanced substantially, opening the door for a more complete and reproducible phylogenetic dataset. At a global scale, there exist recent phylogenies, but they are inevitably highly incomplete for any given region. When tested for the Dutch Flora, the global phylogeny of Jin et al. (2026) showed substantial incongruences (32 cases), including species placed in incorrect phylogenetic positions, making it unsuitable for fine-scale regional analyses. This lack of a recent high-resolution and dated phylogeny prevents testing hypotheses about the (even recent) regional evolutionary and biogeographic history of species and their traits, thereby preventing the valorisation of the massive information already available on traits and distributions of species across the Netherlands and much of oceanic low-altitude Western Europe.

Our objectives are threefold: (i) to assemble and curate a taxonomically consistent species list of native Dutch angiosperms; (ii) to reconstruct a reproducible molecular phylogeny using a multi-locus dataset and state-of-the-art phylogenetic methods; and (iii) to make the resulting trees, alignments, and metadata openly available for use in biodiversity, ecological, and conservation research. By offering this updated and transparent phylogenetic framework, we aim to facilitate a broad range of studies—from quantifying phylogenetic diversity in Dutch ecosystems and conserving evolutionary heritage in the region to placing local biodiversity patterns in a wider evolutionary context. Native-only phylogenies remain fully appropriate for studies of deep-time evolutionary processes contributing to building up the regional species pool, phylogenetic diversity of vegetation types and biogeographic patterns, as neophytes have been introduced too recently to be relevant at evolutionary timescales. However, we acknowledge that analysis of present processes driving phylogenetic diversity of local communities would benefit from also including non-native species.

## General description

### Purpose

The objective of this research is to provide an up-to-date and taxonomically standardized phylogenetic tree of the native angiosperm flora of the Netherlands, resolved as a maximum clade credibility tree and time-calibrated.

## Sampling methods

### Study extent

**Figure 1.**
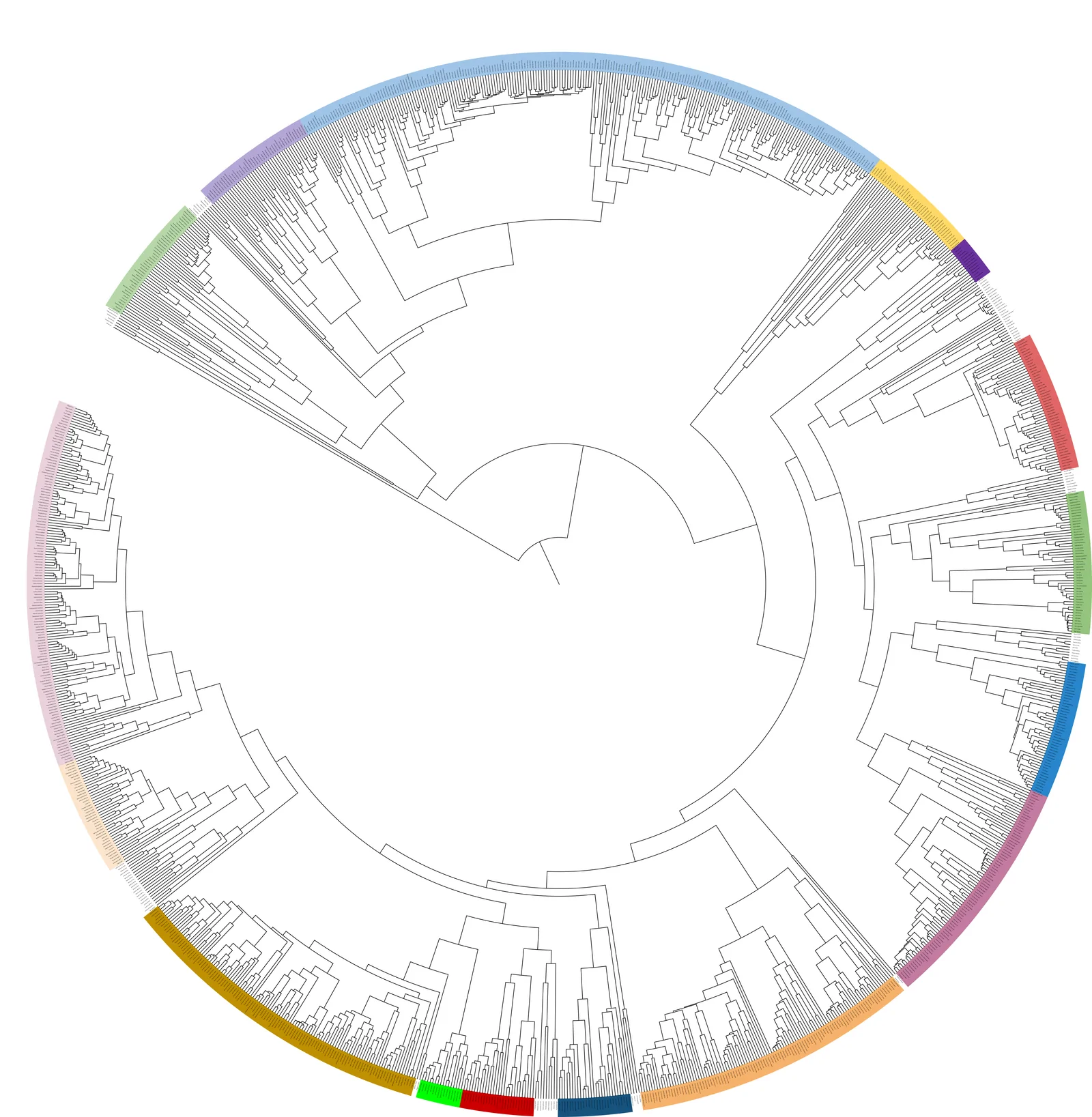
Time-calibrated phylogenetic tree of the Dutch angiosperm flora (n = 1178) constructed from BEAST analysis. This tree was selected as an illustration for its high level of resolution (99.92%). Colors indicate the major plant orders: light green = Alismatales; light purple = Asparagales; light blue = Poales; yellow = Ranunculales; purple = Saxifragales; light red = Brassicales; green = Malpighiales; blue = Fabales; pink = Rosales; orange = Caryophyllales; dark blue = Ericales; dark red = Gentianales; chartreuse = Boraginales; gold = Lamiales; eggshell = Apiales; light pink = Asterales.

The dataset covers the native angiosperm flora of the Netherlands (which is also core to the flora of adjacent countries). Species records were compiled from the most recent SynBioSys (Hennekens and Schaminée 2002) species list, cross-checked against the World Checklist of Vascular Plants (Govaerts et al. 2021), and matched to publicly available DNA sequences from GenBank (Sayers et al. 2021). Molecular data include the nuclear ribosomal ITS region and at least one plastid marker (*matK*, *rbcL*, *trnL–trnF*, or additional loci when available). The dataset includes 1178 species (Fig. 1) and provides a comprehensive and reproducible phylogenetic framework for biodiversity research in the Netherlands.

### Sampling description

No field sampling was conducted.

### Quality control

Several steps were taken to ensure the reliability of the dataset. Taxonomic consistency was verified by matching all species names to the World Checklist of Vascular Plants (Govaerts et al. 2021), which reduces the risk of synonymy or misapplied names. Non-native taxa, hybrid species, and records that could not be confidently assigned to species were excluded, ensuring that the dataset reflects only the native Dutch flora. A species was considered native if reported as native by either the Plant of the World Online (POWO 2026) (i.e., present before the last Ice Age or arrived by natural colonisation) or Euro+Med databases (Euro+Med 2026) (i.e., introduced and present before 1500). Species reported as introduced post 1500, introduced, or cultivated in either database were excluded. Hybrid taxa were identified and excluded based on the SynBioSys (Hennekens and Schaminée 2002) species list.

For the molecular data, we relied exclusively on sequences deposited in GenBank (available in the present dataset), prioritizing those that were complete and taxonomically verified. Multiple sequences for the same taxon were checked for consistency, and cases of apparent misidentification or poor sequence quality were discarded. We selected one of the most complete and informative sequences per gene per species. Alignments were inspected and manually adjusted where necessary to preserve positional homology.

Phylogenetic analyses were run with widely used, peer-reviewed software (BEAST v10.5.0 (Suchard et al. 2018) and MrBayes v3.2.7 (Ronquist et al. 2012) under partitioned substitution models.

Outliers (e.g., unusually long branches) were flagged during tree inspection. Posterior probabilities from BEAST and MrBayes were verified for consistency with replicate runs. Although separate analyses of nuclear and plastid markers would be desirable, the scale of this study and its primary purpose of enabling phylogenetic diversity analyses justify the use of concatenated datasets. Nodes with weak statistical support may reflect potential reticulate evolution and are flagged for future detailed analyses. For each subtree, a single calibration point was applied: the estimated root age of each clade as inferred by Bartish et al. (2016), which itself was derived Hermant et al. (2012). This root age was used as a prior in each phylogenetic analysis. The exact calibration priors for each subtree are fully documented in the 36 MrBayes and BEAST input files provided in the dataset. A summary of the root age priors used for each of the 36 subtrees is provided in Supplementary Table S1 (Suppl. material 1).

### Step description

**Figure 2.**
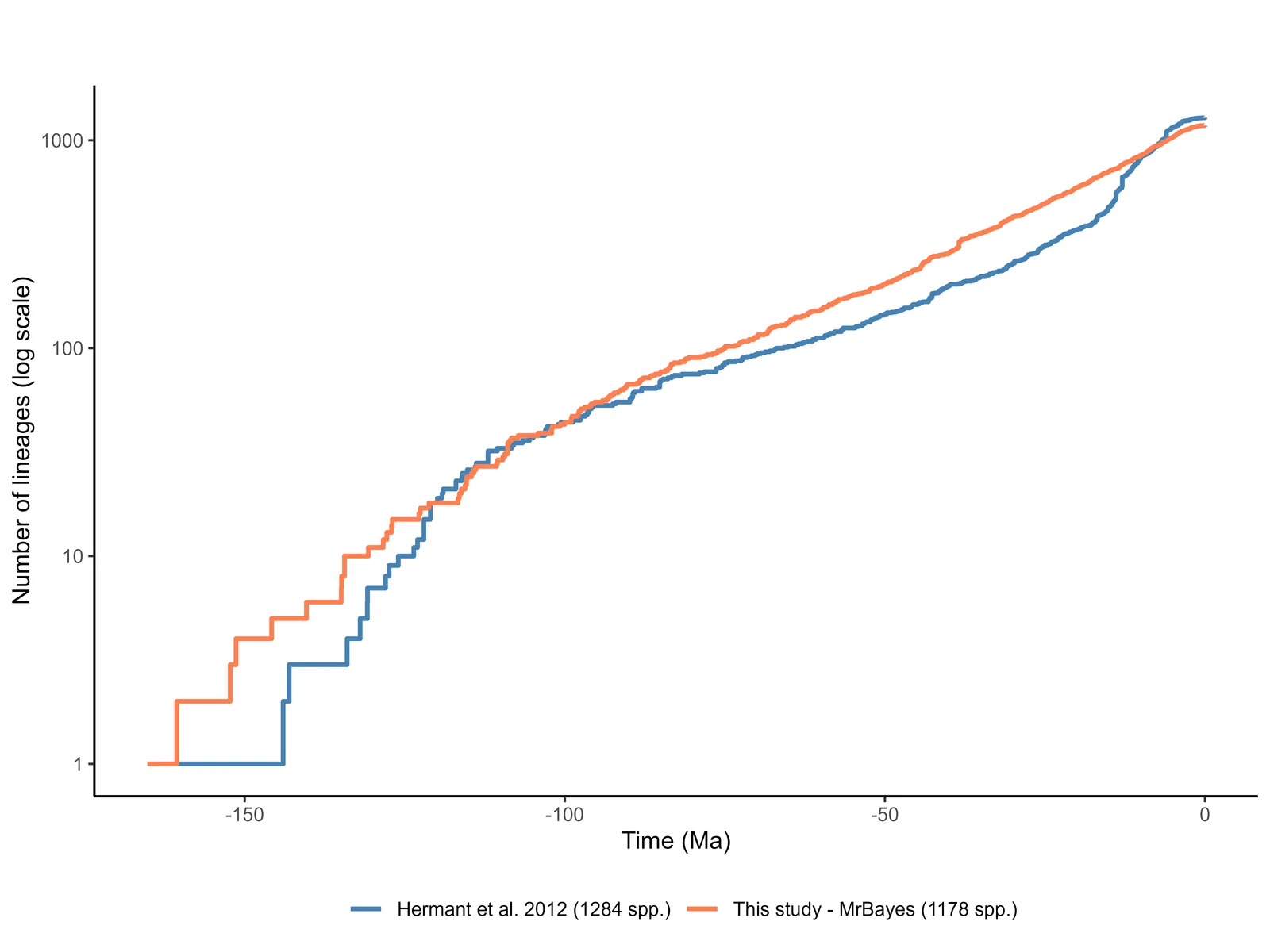
Comparison of lineage-through-time plots based on phylogenies of Hermant et al. (2012) (blue) and our study (orange).

Species lists were retrieved from the most up-to-date list of SynBioSys (Hennekens and Schaminée 2002), and exotics and hybrid species were removed. Species names were then updated to the World Checklist of Vascular Plants (Govaerts et al. 2021), using the R package "U.Taxonstand" (Zhang and Qian 2023). This package automatically resolves synonyms and misspelled names by matching all species names to WCVP counterparts. In practice, as we relied on recent and high-quality GenBank (Sayers et al. 2021) sequences, such conflicts in nomenclature were rare (70 cases) and were resolved manually by using POWO (POWO 2026). Molecular sequence data were retrieved for 2 to 6 genes for each plant species from GenBank (Sayers et al. 2021), depending on their availability in GenBank. Phylogenetic analyses were conducted on concatenated multi-locus datasets combining the nuclear *ITS* region with at least one plastid marker (*matK, trnL–trnF, psbH–trnA*, or *rbcL*). Individual gene alignments were concatenated and partitioned by gene combination to account for locus-specific substitution patterns using MEGA 11.0.13 (Tamura et al. 2021). To improve resolution across the Dutch angiosperm flora and to provide calibrations for basal nodes of the tree based on literature, trees were first inferred for individual taxonomic groups (orders and families) using MrBayes (Ronquist et al. 2012), as described in Hermant et al. (2012). The approach is based on the creation of subsets of closely-related taxa at the level of orders or families. Our objective was to keep the number of subsets to a minimum and, at the same time, to reduce the proportion of missing data in the alignments. These objectives were sometimes contradictory, so there was a trade-off between the size of the subset and the number of genes included into analyses. The optimal size of a subset was between 50 and 100 taxa, but we also created several subsets with considerably smaller sizes (5-10 taxa). In BEAST analyses of separate clades (orders, families, and genera), input files were prepared in BEAUti (Suchard et al. 2018), with substitution models assigned per partition: GTR + Gamma for *ITS* + *matK*, GTR + Gamma + Invariant sites (I) for *ITS* + *psbH–trnA*, and TN93 + Gamma for *ITS* + *trnL–trnF*. For each clade analysis, one or more species from a closely related family or genus were included as an outgroup to root the tree. Analyses were run under an uncorrelated relaxed molecular clock (Drummond et al. 2006) and a Yule speciation process (Yule 1925). Posterior distributions were summarized using TreeAnnotator v10.5.0 (Suchard et al. 2018), producing maximum clade credibility (MCC) trees with mean node heights and posterior probabilities annotated. The individual subtrees were then merged into a single, comprehensive phylogeny in R using the packages ape (Paradis and Schliep 2018), geiger (Pennell et al. 2014), and phytools (Revell 2024), producing a tree representing the entire native Dutch angiosperm flora. The 36 subtrees were defined based on taxonomic ranks (orders, families, or genera), with the level of subdivision determined by clade size. Large orders were subdivided into families or genera when necessary to keep analyses computationally tractable, while smaller clades were sometimes analyzed at the family or genus level directly. Although inferring the entire tree as a single unit would be possible with alternative approaches, the subtree inference and merging strategy was preferred as it allowed clade-specific calibration priors and substitution models to be applied, and follows the approach of Hermant et al. (2012). Following the merging step, each subtree was rescaled to match the age of its corresponding node in the global tree using the "rescale() function (model = 'depth')" from the geiger package (Pennell et al. 2014), which proportionally adjusts all branch lengths within the subtree to fit the target root age. Rescaled subtrees were then attached to the global tree using the "bind.tree()" function from phytools (Revell 2024), ensuring temporal consistency and ultrametricity across all clades. The five species lacking sufficient molecular data were reinserted at the appropriate genus level to maximize taxonomic coverage while maintaining phylogenetic consistency. For comparison, a second phylogeny was constructed using MrBayes v3.2.7 (Ronquist et al. 2012) with the same concatenated alignments and partitioning scheme, providing an independent Bayesian framework for evaluating tree topology. Compared to Hermant et al. (2012), which has a resolution of 79.24% (153 polytomous nodes), our MrBayes phylogeny showed an overall resolution (93.09% of nodes resolved; 95.32% excluding the incompletely resolved genus *Rubus*) and the BEAST phylogeny (97.84%; 99.91% excluding Rubus), further justifying the use of the BEAST maximum clade credibility tree as the primary phylogeny. In the BEAST-based phylogeny, 67.9% of nodes have a posterior probability above 0.95 and 92.3% above 0.50. The LTT plot (Fig. 2) further illustrated the improved resolution of the present phylogeny, showing a smoother and more continuous accumulation of lineages through time compared to the stepped pattern observed in Hermant et al. (2012).

All BEAST and MrBayes NEXUS files correspond to the exact analyses used to generate the published trees and contain the full model specifications and priors required to reproduce the results.

## Geographic coverage

### Description

Netherlands

## Taxonomic coverage

### Description

This dataset includes all native angiosperm species recorded in the Netherlands according to the latest SynBioSys (Hennekens and Schaminée 2002) species list. It encompasses 1178 species across 92 families and 481 genera, spanning the major orders of flowering plants as classified by APG IV (The Angiosperm Phylogeny Group 2016) and illustrated in Fig. 1. A notable exception is the genus *Rubus*, which is highly diverse in the Netherlands but for which DNA sequences were only available for 36 species in GenBank; at least 26 additional native *Rubus* species could not be included due to the lack of publicly available molecular data, representing the most significant gap in taxonomic coverage of this dataset. This limitation reflects the complex taxonomy of this apomictic genus, for which molecular data remain scarce in public databases. The complete list of species included in the phylogeny is available in the dataset via the GlobalTree file (which contains all tip labels) and the DutchAngiospermGBcodes CSV file (which links each species to its GenBank accession numbers and vouchers), both accessible on iDiv (https://doi.org/10.25829/idiv.3600-myr3t3)

## Usage licence

### Usage licence

Creative Commons Public Domain Waiver (CC-Zero)

### IP rights notes

The phylogenetic dataset is stored in iDiv (https://doi.org/10.25829/idiv.3600-myr3t3) and is currently under embargo. It will be made publicly accessible under a CC-BY 4.0 license once the data paper has been accepted.

## Data resources

### Data package title

Molecular phylogenetic dataset of the native angiosperm flora of the Netherlands

### Resource link

https://doi.org/10.25829/idiv.3600-myr3t3

### Number of data sets

6

### Data set 1.

#### Data set name

BeastInput 

#### Description

36 TXT files containing the input files for the Beast analysis.

### Data set 2.

#### Data set name

BeastTrees

#### Description

Two folders containing the 36 TXT files containing the output trees from the Beast analysis, both in Newick (36 files) and in Nexus (36 files).

### Data set 3.

#### Data set name

MrBayesInput

#### Description

36 TXT files containing the input files for the MrBayes analysis.

### Data set 4.

#### Data set name

MrBayestree

#### Description

Two folders containing the 36 TXT files containing the output trees from the MrBayes analysis, both in Newick (36 files) and in Nexus (36 files).

### Data set 5.

#### Data set name

GlobalTree

#### Description

2 TXT files containing the global Dutch angiosperm phylogeny from both Beast analysis (1 file) and MrBates (1 file).

### Data set 6.

#### Data set name

DutchAngiospermGBcodes

#### Description

CSV file containing all the GenBank codes and voucher used for each species.

## Supplementary Material

A2D40CB2-D6F1-5E86-9A62-04210A3A00E4 10.3897/BDJ.14.e192282.suppl1 Supplementary material 1 Posterior probabilities and node ages estimates Data type Phylogenetic values Brief description Posterior probabilities and the nodes ages estimates for each nodes in the BEAST and MrBayes trees. File: oo_1664364.xlsx https://binary.pensoft.net/file/1664364 Leclère, T., Prinzing, A., Gerhold, P. & Bartish, I.
